# Severe erytrodermic psoriasis and arthritis as clinical presentation of a CARD14-mediated psoriasis (CAMPS)

**DOI:** 10.1186/1546-0096-13-S1-P57

**Published:** 2015-09-28

**Authors:** S Signa, M Rusmini, E Campione, I Gueli, A Grossi, A Omenetti, L Bianchi, A Martini, I Ceccherini, M Gattorno

**Affiliations:** 1 IRCCS G. Gaslini, U.O. Pediatria II, Genoa, Italy; 2 IRCCS G. Gaslini, U.O Genetica Medica, Genoa, Italy; 3 Tor Vergata University of Rome, Department of Dermatology, Rome, Italy

## Introduction

Autosomal dominant gain of function mutations in caspase recruitment domain family member 14 (CARD14) were found to cause plaque psoriasis in two families and severe generalized pustular psoriasis as a monogenic form of childhood (CARD14-mediated psoriasis, CAMPS) [1]. CARD14 mutations have also been implicated in plaque-type psoriasis and pityriasis rubra pilaris [2].

## Objectives

Describing a family with an unusual clinical phenotype characterized by some members with childhood-onset erytrodermic psoriasis first localized and then diffuse over all the skin surface; in some family members is also reported psoriatic arthritis.

## Patients and methods

We assessed for the first time the family in december 2013, because of their skin lesions and family recurrence for erytrodermic psoriasis associated to arthritis in some cases. There are three pairs of twins, five of them presenting psoriasis and two of them presenting psoriatic arthritis. The children presented poor clinical response to topic and systemic therapy with antihistamine, steroid, retinoids, cyclosporine and etanercept. After exclusion of the most common genes associated to autoinflammatory diseases (IL36RN, IL1RN, MVK, TNFRSF1A, NLRP3, NLRP12, MEFV, IL1RN, NOD2, PSMB8, PSTPIP1, LPIN2) we approached the new gene search by subjecting to Whole Exome Sequencing (WES) analysis five members of the family. Samples were processed in outsourcing and raw data were transferred to our lab for the bio-informatic analysis.

## Results

Among variants shared by the four affected individuals and not present in the unaffected subject, a missense mutation of the CARD14 gene resulted worth of further investigation. In particular, it was the case of an exon4 heterozygous nucleotide change, c.446T>G, leading to the missense amino acid substitution p.L149R. The presence of this variant was validated by Sanger sequencing not only in the affected members undergone WES, but also assessed in all the rest of the available family members. This allowed us to confirm the expected segregation of the CARD14 mutation with the disease phenotype.

## Conclusions

CARD14 gain of function mutations can give rise to unusual clinical phenotype like diffuse erytrodermic psoriasis and psoriatic arthritis. WES appears to be a powerful, suitable and proper approach to identify new SAID genes, thus also disclosing new molecular pathogenic mechanisms.
